# Global mental health: good news, bad news and responsibilities for the future

**DOI:** 10.1017/S2045796019000738

**Published:** 2019-11-19

**Authors:** C. Barbui, E. Albanese

**Affiliations:** 1 Department of Neuroscience, Biomedicine and Movement Sciences, Section of Psychiatry, World Health Organization Collaborating Centre for Research and Training in Mental Health and Service Evaluation, University of Verona, Verona, Italy; 2 Department of Psychiatry, Faculty of Medicine, World Health Organization Collaborating Centre for Research and Training in Mental Health, University of Geneva, Geneva, Switzerland; 3 Faculty of Bio-Medical Sciences, Università della Svizzera Italiana, Lugano, Switzerland

**Keywords:** Epidemiology, health service research, mental health, psychiatric services, quality of care

At a global level considerable progress has been made over the past few decades on research, policy and implementation activities that are specifically relevant for mental health in resource-poor settings. In 2018, the Lancet Commission on global mental health and sustainable development provided an historical overview of this progress, reframed the concept of mental health by bringing together knowledge from diverse scientific perspectives and real-world experiences, and called for urgent action to translate current knowledge into concrete activities, aiming to promote mental health, prevent mental disorders and scale up services to detect, treat and support recovery of people with mental disorders (Patel *et al*., 2018).

Against this background, in June 2019 a symposium was organised during the annual congress of the European Network for Mental Health Service Evaluation in Lisbon, Portugal. The aim was to present a visionary reflection on research, policy and implementation priorities for the global mental health agenda of the next decade. Three speakers kindly accepted to contribute, addressing the following four questions:

1. What are the priorities for the next ten years of *research* in global mental health?
2. What are the priorities for the next ten years of *policy activity* in global mental health?
3. What are the priorities for the next ten years of *implementation activities* in global mental health?
4. Should research, policy and implementation be given similar relevance or, rather, should any of the three activities be given priority over the others?

The symposium was so successful, participatory and challenging that we thought it was important to publish its main reflections and considerations as Editorials in *Epidemiology and Psychiatric Sciences* (Caldas de Almeida, 2020; Lund, 2020; Saraceno, 2020). We suggest readers to go through the Editorials attempting to group the points raised by the authors into the following three categories.

First, there is good news. Global mental health has registered a gigantic growth during the last decade. It has been quite successful in attracting the interest of academia, which has activated a number of training initiatives such as summer courses, masters and diplomas, and the interest of funders. Funding schemes focused on topics related to global mental health multiplied and contributed to the exponential increment in research outputs, many of which extremely innovative. Also through this robust evidence, the discipline of global mental health now provides regular support to policy making activities and it has a visible, legitimate and recognised position within global health. Moreover, the global mental health movement, which entails important sectors in society beyond academia and health, maintains and effectively promotes the notion of mental health not only as a fundamental human right (Caldas de Almeida, 2020; Lund, 2020; Saraceno, 2020), but also as a global public good (Patel *et al*., 2018), providing unprecedented opportunities to a central positioning of mental health in both the global health and the sustainable development agendas.

Second, there is bad news. As the definition and content of global mental health is still unclear, there is a risk of an unbalance between a disproportionate focus on treatment rather than on promotion, prevention and care; a risk of an excessive influence of a western culture implementing a biomedical approach; a risk of an unbalance between an excessive focus on common mental disorders rather than on severe mental disorders where human right violations are still frequent; a risk of poor attention to the translation of national mental health policies into concrete implementation programmes and a risk of an uncritical focus on scaling up of treatments without considering first the development and/or reform of health systems, services and an appropriate infrastructure for such treatments. Additionally, local contexts are often not considered, as if being ‘global’ implies the lack of attention to local social and economic aspects that may act as determinants of mental health (Caldas de Almeida, 2020; Lund, 2020; Saraceno, 2020).

Third, good and bad news may inform future actions, their prioritisation and may additionally shed light on how to untangle the complex interlinks that exist between global mental health challenges. The three editorials identified the following ones: understanding the aetiology of mental health conditions across the life course; addressing the social determinants of mental health; focusing on prevention and promotion interventions; broadening the notion of scaling up of services accounting for local context and human right considerations; understanding the effect of different organisations of mental health care on the mental health and well-being of people with mental disorders; assessing the cost-effectiveness of different models of financing and organising the provision of mental health care and opening a more trans-disciplinary approach, in collaboration with service users and other stakeholders (Caldas de Almeida, 2020; Lund, 2020; Saraceno, 2020).

The three editorials seem to agree on the need of maintaining an open forum for debating the *pros* and *cons* of different strategies that may be activated to produce effective answers to the mental health needs of the population in both high, and low and middle income countries. The three editorials concur that the remarkable advances of the past two decades have greatly contributed to the current connotation of global mental health as an ambitious, goal-oriented discipline, with growing legitimacy in the global health arena. However, several pending challenges remain, including the persistence of barriers for the effective implementation of mental health policies, and the apparent inertness of mental health systems and service reforms. The importance of maintaining an open forum for debate is also highlighted by the salience of mental health for the attainment of sustainable development. Because we maintain that this forum holds the promise to nurture a constructive debate to identify actionable solutions to be addressed and endorsed across sectors, we offer *Epidemiology and Psychiatric Sciences* as one potential platform for such a debate.
